# A river in crisis: water quality, microbial burden, and public health implications of a South African urban river

**DOI:** 10.1128/aem.01566-25

**Published:** 2025-10-08

**Authors:** Sanelisiwe Thinasonke Duze, Luyanda Mkhize, Musa Marimani, Mrudula Patel

**Affiliations:** 1 Department of Clinical Microbiology and Infectious Diseases, Faculty of Health Science, University of Witwatersrand, Parktown 37708 , Johannesburg, South Africa; 2 Department of Anatomical Pathology, Faculty of Health Science, University of Witwatersrand, Parktown 37708 , Johannesburg, South Africa; 3 National Health Laboratory Services, Infection Laboratory Services Laboratory, Parktown 70685, Johannesburg, South Africa; University of Delaware, Lewes, Delaware, USA

**Keywords:** Jukskei River, water quality, feacal contamination, quantitative microbial risk assessment, public health risk

## Abstract

This study evaluated water quality, microbial contamination, and public health implications of a South African urban river. Water samples were collected monthly over 12 months from five sites and analyzed for physico-chemical parameters and microbial indicators (total coliforms and *Escherichia coli*). *Salmonella*, *Shigella,* and *Vibrio cholerae* were enumerated using culture-based methods and confirmed with real-time PCR targeting the *invA*, *ipaH*, and *toxR* genes. The β-Poisson dose-response model estimated the probability of infection (*Pi*) following ingestion of 1 mL, 16 mL, and 37 mL of river water during baptism, swimming by adults and non-adults, respectively. Average values for pH, temperature, and total dissolved solids were 7.29, 18˚C, and 160 mg/L, respectively. The total coliform and *E. coli* counts exceeded 2,000 and 400 CFU/100 mL at most sites. Mean bacterial counts (Log CFU/100 mL) in dry and rainy seasons were 3.22 ± 0.88; 3.28 ± 0.97 for *Salmonella*, 4.60 ± 0.53; 4.45 ± 0.79 for *Shigella,* and 0.73 ± 1.97; 3.27 ± 2.24 for *Vibrio cholerae*. Estimated *Pi* from ingesting 1 mL of water ranged from 2.57% to 24.73, 4.91%–15.99%, and 1.44%–19.5% for *Salmonella*, *Shigella,* and *V. cholerae*, respectively, increasing with the volume ingested and frequency of exposure. Site C, located within a densely populated urban informal settlement, posed the highest infection risk. Our findings highlight the substantial public health risk from this urban river and reinforce the urgent need for improved water quality management and public health interventions to protect vulnerable communities. In addition, results from this study underscore the need for a One Health Approach for enteric pathogen surveillance in South Africa.

**IMPORTANCE:**

Urban rivers in low- and middle-income countries are essential for domestic, religious, and recreational use but often pose significant public health risks. This study quantified microbial contamination and assessed the infection risk from accidental ingestion of water from a South African urban river. Total coliform and *Escherichia coli* counts consistently exceeded safe limits, with high levels of *Salmonella*, *Shigella*, and *Vibrio cholerae*. Infection probabilities of up to 25% for a single exposure, ingesting 1 mL of river water, were noted, which increased with ingested volume and multiple exposures. Risk of infection was higher at sites alongside informal settlements, highlighting the impact of socioeconomic inequities and poor sanitation infrastructure. These findings underscore the urgent need for improved water quality management and integrated One Health surveillance of enteric pathogens to mitigate exposure risks and safeguard vulnerable populations.

## INTRODUCTION

Rivers have played a vital role in the socio-economic development of humans, as sources of drinking water, fisheries, and means of transport. They continue to be crucial in the present day, supporting irrigation in the agricultural sector, domestic and recreational activities, as well as maintaining healthy ecosystems while promoting biodiversity (1, 2). In 2024, the United Nations World Water Development Report reported an increase in global freshwater use, with an average rise of 1% each year since the 1980s (3). Despite this increase in use, water quality faces significant challenges due to industrialization, agricultural production, climate change, population growth, and urbanization, putting pressure on an already scarce resource (4).

The release of untreated sewage, chemical effluents, petroleum leaks, spills, dumping of waste, and agricultural runoff all contribute to river degradation and pollution (4). These water pollutants make river water unusable, resulting in water scarcity, particularly in developing countries like South Africa. Concerningly, contaminated water acts as a reservoir and vehicle for the transmission of pathogenic waterborne microorganisms. Physicochemical parameters such as pH, dissolved oxygen, turbidity, and dissolved solids are commonly used in water quality monitoring (5, 6). In addition, fecal coliforms, particularly *Escherichia coli*, are used as bacterial indicators of fecal contamination (7, 8). The presence of *E. coli* in water indicates the potential presence of other bacterial pathogens, including pathogenic strains of *E. coli* (9).

Waterborne pathogens are responsible for a significant burden of disease, particularly in developing countries (3). Humans can be exposed to waterborne pathogens through multiple routes, including the ingestion of contaminated water, recreational contact with contaminated surface water, food consumption, particularly produce irrigated or washed using contaminated water, and inhalation of aerosolized wastewater (10). Diarrhea is a common symptom of gastrointestinal infections and is the most prevalent water-related disease associated with contaminated water (4). In 2026, water, sanitation, and hygiene (WASH) reported that approximately 829,000 deaths were attributed to diarrheal diseases linked to unsafe WASH practices. The majority of the fatalities were minors aged below five years, constituting 5.3% of the total deaths in this age bracket (11).

In South Africa, diarrheal diseases are notifiable, and surveillance for enteric pathogens is active. However, current surveillance efforts are largely limited to clinical samples, often overlooking environmental sources such as river water, despite their potential role in ongoing transmission. This study aimed to evaluate the water quality and the extent of microbial contamination in an urban South African river and determine the public health risk associated with exposure to enteric pathogenic bacteria in the water.

## MATERIALS AND METHODS

### Study site: Jukskei River

**Fig 1:**
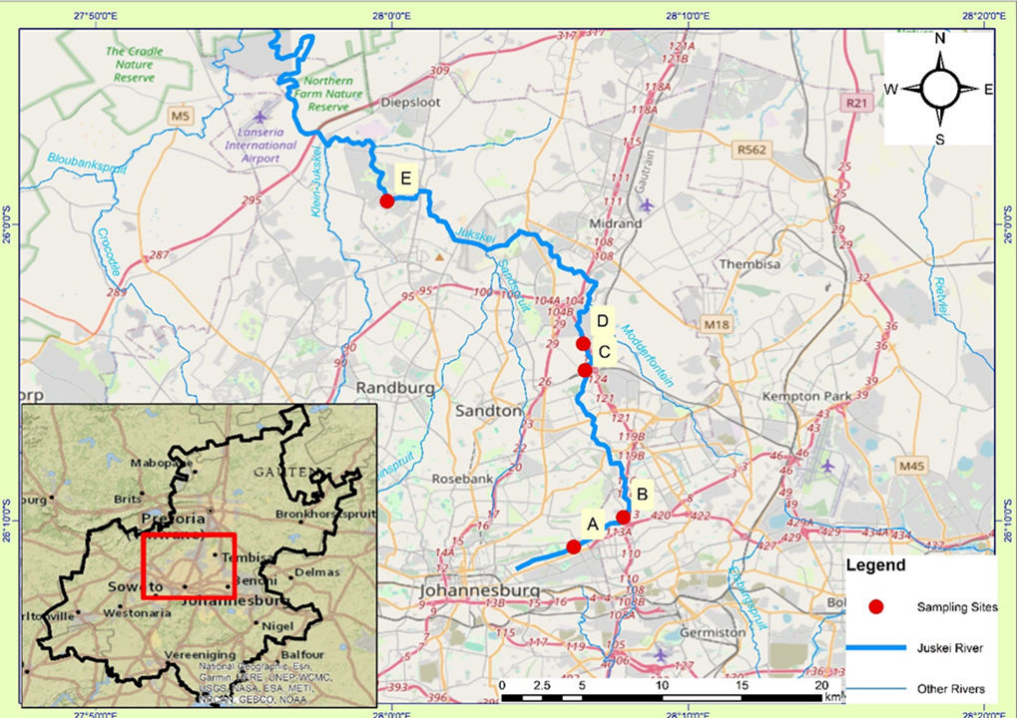
Map showing the five sampling sites A–E (red dots) along the Jukskei River (blue line) in Johannesburg, South Africa. Site A (business district): 26,18147°S, 28,10237°E, site B (recreational park): 26,16475°S, 28,13029°E, site C (densely populated informal settlement): 26,08208°S, 28,10873°E, site D (residential suburb): 26,06715°S 28,10767°E, and site E (Golf Estate): 25,98690°S, 27,99731°E. The map was created using ArcGIS software.

The Jukskei River is one of the largest rivers in Johannesburg, South Africa. The river flows through the vicinity of the Bruma Lake and swings in a northerly direction past recreational parks, formal and informal settlements, and a Golf Estate before discharging to the Crocodile River, which feeds into the Hartbeespoort Dam (Fig. 1). Five distinct sites were identified along the Jukskei River for sample collection. These sites were selected based on surrounding developments, industrial activities, and population density to capture environmental differences (Table S1).

### Sample collection and physicochemical parameters

Water samples were collected once a month from each of the 5 sampling sites over 12 months using sterile 2 L bottles (*n* = 60). During each sampling event, the temperature at the collection point was measured using a portable thermometer. After collection, the samples were stored in cooler boxes with ice and transported to the Department of Clinical Microbiology and Infectious Diseases, University of the Witwatersrand. A 100 mL aliquot of each water sample was sent to the School of Chemistry in the Department of Environmental and Analytical Chemistry at the University of the Witwatersrand for physicochemical parameter analysis. The water quality parameters analyzed included pH and total dissolved solids (TDS, mg/L).

### Total coliforms and *E. coli*

The Quanti-Tray/2000 (IDEXX, Westbrook, ME, USA) was used for the enumeration of Total coliforms (TC) and *E. coli*, following the manufacturer’s instructions. One Colilert-18 sachet was added to a 100 mL water sample and allowed to dissolve at room temperature. The sample was then poured into the Quanti-Tray/2000 (IDEXX, Westbrook, ME, USA), which was sealed using a Quanti Sealer (IDEXX, Westbrook, ME, USA) and incubated for 18 h at 35°C. After incubation, the Quanti-Tray/2000 was examined under 366 nm ultraviolet light. Wells that turned yellow and fluoresced were counted as *E. coli* positive (IDEXX, Westbrook, ME, USA). The most probable number (MPN) of coliforms and *E. coli* was determined using the IDEXX MPN Generator software version 1.4.4 (12) based on the wells showing yellow coloration and blue-white fluorescence, respectively, and the counts were reported as colony-forming units per milliliter (CFU/100 mL).

### Detection and enumeration of enteric bacterial pathogens

One liter of water samples was filtered through a 0.45 µm pore size sterile membrane filter (Labex, South Africa). The filters were aseptically transferred to a 50 mL centrifuge tube (Separations, South Africa) containing 10 mL of phosphate-buffered saline (PBS, pH 7.0) (Merck, Darmstadt, Germany). The bacteria were dislodged from the membrane by scraping with sterile inoculation loops, followed by vortexing for 1 min to ensure thorough resuspension. Subsequently, 10-fold serial dilutions were prepared from the resuspended samples using sterile PBS and 100 µL from each dilution were inoculated onto selective agar plates, including Brilliance *Salmonella* Agar (Thermo Fisher Scientific, MA, USA) for *Salmonella* sp., Xylose Lysine Deoxycholate (XLD) agar (Thermo Fisher Scientific, MA, USA) for *Shigella* sp., and Thiosulfate Citrate Bile-Salts Sucrose (TCBS) Agar (Thermo Fisher Scientific, MA, USA) for *Vibrio* sp. All inoculated plates were incubated at 37°C for 24 h.

Presumptive colonies were identified based on characteristic morphology, with *Salmonella* appearing as purple colonies on Brilliance *Salmonella* Agar, *Shigella* as red on XLD, and *Vibrio* as yellow colonies on TCBS. Colonies were enumerated from plates containing 30-300 CFU, and results were expressed as log CFU/100 mL. Enumeration was performed from the dilution level that yielded visible and countable colonies within this range. For molecular confirmation, one to two presumptive colonies of each target organism were randomly selected per sampling site. These colonies were sub-cultured onto 5% blood agar (Thermo Fisher Scientific, MA, USA) and incubated at 37°C for 24 h prior to polymerase chain reaction (PCR) analysis.

### Identification of *Salmonella*, *Shigella*, and *Vibrio cholerae*

Pure colonies from the 5% blood agar (Thermo Fisher Scientific, MA, USA) plates were subjected to genomic DNA extraction using the boiling method, followed by PCR for confirmation. Briefly, 400 µL of Tris-EDTA (TE) buffer (Merck, Darmstadt, Germany) was transferred into a 1.5 mL Eppendorf tube. A loopful of culture was added to the TE buffer, vortexed, and boiled at 95°C for 25 min in a heating block. The sample was then centrifuged at 12,000 × *g* for 3 min (Eppendorf centrifuge 5417C, Merck, NJ, USA). Subsequently, 20 µL of the supernatant was transferred to a new 1.5 mL Eppendorf tube containing 80 µL of TE buffer, vortexed, and centrifuged at 12,000 × *g* for 1 min. Real-time PCR assays were optimized for *Salmonella*, *Shigella,* and *V. cholerae* identification, using primer pairs listed in Table 1. A total reaction volume of 20 µL, comprised of 2 × PowerTrack SYBR Green Master Mix (Thermo Fisher Scientific, MA, USA), 0.5 µM of each primer, 7 µL nuclease-free water, and 2 µL of template DNA, was used for all amplifications.

**TABLE 1:** Target genes and primer sequences used for the detection of *Salmonella* sp., *Shigella* sp., and *V. cholerae*

Pathogen	Target gene	Primer sequence (5′–3′)	Product size (bp)	Reference
*Salmonella*	*invA*	F: GTGAAATTATCGCCACGTTCGGGCAA R: TCATCGCACCGTCAAAGGAACC	284	(13)
*Shigella*	*ipaH*	F: CCT TGA CCG CCT TTC CGA TA R: CAG CCA CCC TCT GAG GTA CT	606	(14)
*V. cholerae*	*toxR*	F: TGG CAT CGT TAG GGT TAG CAA R: CAT TCA CAG CCC TGA AGT TTC A	68	(15)

Amplification reactions were performed in a Roche LightCycler 480II instrument (Roche Diagnostics, Germany). The cycling conditions included an initial enzyme activation step of 95°C for 2 min. This was followed by 40 cycles of denaturation at 95°C for 2 min, followed by variable annealing specific for each gene and an elongation step at 72°C for 1 min. The annealing conditions were as follows: *InvA* gene at 60°C for 30 s, *ipaH* gene at 55°C for 30 s, and *toxR* gene at 55°C for 1 min. Fluorescence intensity was measured at each PCR cycle, and crossing-point (Cp) analysis was used to determine the cycle at which target gene amplification became detectable. Cp values were automatically calculated using the LightCycler 480 software. Melting curve analysis was performed post-amplification to confirm specificity, with single, sharp peaks indicating successful and specific amplification. A positive control and a no-template control were included in each reaction for quality assurance. The positive controls used in each PCR were *Salmonella enterica* subspecies *enterica* serovar Typhimurium ATCC 14028, *Shigella flexneri* ATCC 12022, and *Vibrio cholerae* NCTC 8021.

### Quantitative microbial risk assessment

To assess the health risk associated with exposure to *Salmonella, Shigella*, and *Vibrio cholerae* in the Jukskei River, the beta (β)-Poisson response model was used. The beta-Poisson model captures the heterogeneity in host-pathogen interactions and is considered a suitable fit for *Salmonella* sp., *Shigella* sp., and *Vibrio cholerae* (16, 17). The probability of infection (*Pi*) was estimated based on three scenarios: (i) a baptism scenario involving accidental ingestion of 1 mL of water during a full-body immersion baptism; (ii) an adult swimming scenario with ingestion of 16 mL of water, based on average ingestion volumes reported for adults during a 45 min recreational swim (18); and (iii) a non-adult swimming scenario involving ingestion of 37 mL of water, based on average ingestion volumes for non-adults during swimming activities (18). Due to the absence of scientific data on water ingestion during baptisms, the 1 mL volume was assumed by adjusting adult swimmer ingestion volumes downward to reflect the typically shorter and often controlled nature of baptisms. The exposure dose of pathogens was determined using Equation 1 (Eq.1) adapted from Nkwatoh et al. (19). The daily *Pi* for each scenario was determined for each sample using Equation 2 (Eq. 2), and the cumulative annual risk of infection, assuming weekly exposure (*n* = 52), was calculated using Equation 3 (Eq. 3).


(1) N=Volume ingested (mL) x Mean bacterial counts (CFU/100 mL)/100

(2) Pi=1−(1+(N/β)−α

(3) $$Pi_{annual}=1-(1-Pi{)}^{n}$$

Where *N* is the dose ingested, *α* and *β* are the organism-specific equation parameters, which are *α* = 0.21; *β* = 49.78 for *Salmonella* sp., *α* = 0.265; *β* = 1,480 for *Shigella* sp., and *α* = 0.250; *β* = 243 for *Vibrio cholerae*, based on previously published parameters (19–22), and lastly, *n* is the number of times exposure occurs (*n* = 52).

### Data analysis

All data were collected and captured using Microsoft Excel 2010 data software. For bacterial enumeration, statistical analysis was performed using a parametric test with StataNow/SE version 18.5 (Stata Software, Texas, USA), as the data were normally distributed. Bacterial counts from the quantitative analysis were expressed as mean ± standard deviation (SD), and differences in bacterial counts between the sampling sites were assessed by one-way ANOVA followed by a pairwise analysis using Tukey’s Test to find means that are significantly different from each other. The differences in bacterial counts between rainy and dry seasons were assessed using a paired *t*-test. Data were regarded as statistically significant when *P* < 0.05.

## RESULTS

### Water quality of the river

The water quality of the river was surveyed over a year-long period across five sites. Overall, the mean pH of the water samples ranged from 7.02 at site C to 7.56 at site E. The temperature varied between 19°C and 22°C during the rainy season, and 14°C to 17°C in the dry season (Table 2). The mean TDS of water samples was lowest in site A at 133 mg/L and highest at site D at 197 mg/L (Table 2). Total coliform and *E. coli* counts across the sampling sites and seasons were far above the acceptable limits. TC counts ranged between 1,769 and >2,420 CFU/100 mL during the rainy season and from 1,809 to >2,420 CFU/100 mL during the dry season. On the other hand, *E. coli* counts ranged between 141 and 1,737 CFU/100 mL during the rainy season and 213 and 2,131 CFU/100 mL during the dry season. The highest TC and *E. coli* counts were observed in sites C and D, whereas the lowest TC and *E. coli* counts were noted in site E (Table 2).

**TABLE 2:** Summary of physico-chemical parameters, total coliform (TC), and *E. coli* counts across the five sampling sites^*a*^

Site	Season^*b*^	Mean ± SD (January to December 2023)				
		pH	Temp (°C)	TDS^*d*^ (mg/L)	TC (CFU/100 mL)	*E. coli* (CFU/100 mL)
A	Rainy (*n* = 6)	7.20 ± 0.29	19 ± 0.98	154 ± 7.98	>2,420 ± 0.00	494 ± 213
	Dry (*n* = 6)	7.07 ± 0.62	14 ± 2.94	112 ± 29.65	2,348 ± 177	417 ± 200
	Total (*n* = 12)	7.14 ± 0.44	17 ± 3.2	133 ± 2.26	2,384 ± 125	455 ± 211
B	Rainy (*n* = 6)	7.04 ± 0.15	19 ± 1.03	113 ± 22.86	>2,420 ± 0.00	484 ± 329
	Dry (*n* = 6)	7.28 ± 0.41	15 ± 3.2	182 ± 7.04	>2,420 ± 0.00	869 ± 672
	Total (*n* = 12)	7.16 ± 0.35	17 ± 3.08	155 ± 19.79	>2,420 ± 0.00	677 ± 543
C						
	Rainy (*n* = 6)	7.03 ± 0.39	20 ± 1.22	194 ± 67.8	>2,420 ± 0.00	1,737 ± 1,058
	Dry (*n* = 6)	7.02 ± 0.1	16 ± 3.56	145 ± 21.91	>2,420 ± 0.00	2,105 ± 755
	Total (*n* = 12)	7.02 ± 0.37	18 ± 3.48	170 ± 70.0	>2,420 ± 0.00	1,896 ± 892
D	Rainy (*n* = 6)	7.00 ± 0.08	20 ± 1.17	178 ± 26.14	>2,420 ± 0.00	1,716 ± 1,092
	Dry (*n* = 6)	7.10 ± 0.27	16 ± 3.26	217 ± 16.52	>2,420 ± 0.00	2,132 ± 706
	Total (*n* = 12)	7.05 ± 0.16	18 ± 3.65	197 ± 43.13	>2,420 ± 0.00	1,924 ± 903
E	Rainy (*n* = 6)	7.82 ± 0.24	22 ± 0.81	196 ± 55.2	1,769 ± 731	141 ± 95
	Dry (*n* = 6)	7.29 ± 0.1	17 ± 3.38	113 ± 8.96	1,809 ± 734	213 ± 221
	Total (*n* = 12)	7.56 ± 0.34	19 ± 4.13	154 ± 21.92	1,789 ± 699	177 ± 167
SAWQ^*c*^		6.0–9.0	18–24	0–450	0–5	0

^*a*^ The values represent the mean ± SD obtained during the rainy and dry seasons in 2023.

^*b*^ Rainy season = September–February; dry season = March–August.

^*c*^ SAWQ = South 228 African Water Quality guideline for domestic use.

^*d*^ The WHO recommended value for TDS (total dissolved solids) is a value in the range of 300–500 mg/L (23).

### Microbial burden in the river

**Fig 2:**
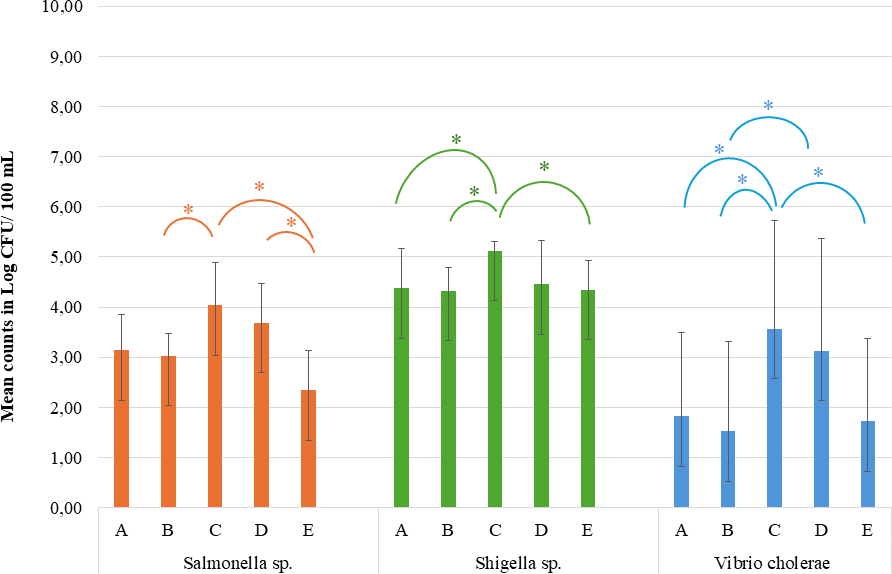
Mean counts for *Salmonella* sp., *Shigella* sp., and *Vibrio cholerae* across the five sampling sites throughout the study period. *Statistically significant between sampling sites when *P <* 0.05*.* For *Salmonella* sp., significant differences were found between site B and C (*P =* 0.014), sites C and E (*P <* 0.001), and sites D and E (*P =* 0.001). For *Shigella* sp., statistically significant differences were between sites A and C (*P =* 0.045), sites B and C (*P =* 0.027), and sites C and E (*P =* 0.034). For *Vibrio cholerae*, significant differences were between sites A and C (*P =* 0.025), sites B and C (*P =* 0.006), sites B and D (*P =* 0.044), and sites C and E (*P =* 0.017).

*Salmonella* sp., *Shigella* sp., and *Vibrio cholerae* were detected in all five sites along the Jukskei River. Raw data is provided in Table S2 in the supplementary material. All the presumptive colonies were positive for the respective targeted genes for *Salmonella* (*invA*), *Shigella* (*ipaH*), and *Vibrio cholerae* (*toxR*). The levels of contamination varied across sampling sites and between seasons. The mean counts of *Salmonella* sp. were 3.28 ± 0.97 Log CFU/100 mL during the rainy season and 3.22 ± 0.88 Log CFU/100 mL in the dry season (Table 3). Although seasonal differences in *Salmonella* levels were not statistically significant, significant differences were observed between sampling sites. Notably, statistically significant differences were found from site to site (*P <* 0.001). Specifically, significant differences were found between site B and C (*P =* 0.014), sites C and E (*P <* 0.001), and sites D and E (*P =* 0.001). The highest *Salmonella* counts were recorded in sites C and D, with the lowest counts observed in site E (Fig. 2). The mean concentrations of *Shigella* sp. were 4.45  ±  0.79 Log CFU/100 mL during the rainy season and 4.61  ±  0.53 Log CFU/100 mL during the dry season, which were notably higher than those recorded for *Salmonella* sp. No significant seasonal differences were observed in *Shigella* sp. counts (Table 3). However, statistically significant differences were observed from site to site (*P =* 0.017), specifically, between sites A and C (*P =* 0.045), sites B and C (*P =* 0.027), and sites C and E (*P =* 0.034) (Fig. 2).

**TABLE 3:** Bacterial counts during the rainy and dry seasons in the river

Organism	Mean ± SD bacterial counts in log CFU/100 mL (*n* = 30)		*P*-value
	Rainy	Dry	
*Salmonella* sp.	3.28 ± 0.97	3.22 ± 0.88	0.3714
*Shigella* sp.	4.45 ± 0.79	4.61 ± 0.53	0.8269
*Vibrio* sp.	3.44 ± 1.92	1.27 ± 1.50	0.0001^*a*^

^*a*^ Statistically significant between rainy and dry seasons when *P* < 0.05.

Compared to *Salmonella* sp. and *Shigella* sp., *Vibrio cholerae* exhibited the lowest mean counts. A statistically significant difference in the *Vibrio* counts was observed between the rainy and dry seasons (*P =* 0.0001), with higher mean counts recorded during the rainy season (3.44  ±  1.92 Log CFU/100 mL) compared to the dry season (1.27  ±  1.50 Log CFU/100 mL) (Table 3). Additionally, significant differences were observed across sampling sites (*P =* 0.002), with notable differences between sites A and C (*P =* 0.025), sites B and C (*P =* 0.006), sites B and D (*P =* 0.044), and sites C and E (*P =* 0.017) (Fig. 2).

### Probability of infection following exposure to the contaminated water

The predicted probability of infection (*Pi*) for *Salmonella* sp., *Shigella* sp., and *V. cholerae* under the three exposure scenarios is shown in Table 4. Assuming a single ingestion of 1 mL of contaminated river water during a full-body immersion baptism, *Pi* ranged from 2.57% to 27.73% for *Salmonella* sp., 4.91% to 15.99% for *Shigella* sp., and 1.44% to 19.5% for *V. cholerae* (Table 4). For a single exposure to 16 mL and 37 mL of water by adult and non-adult swimmers, respectively, the *Pi* remained below 50% at most sites. However, swimming in site C poses an even greater risk as *Pi* exceeded 50% for both *Salmonella* sp. (50.78% and 57.89%) and *Shigella* sp. (51.33% and 60.58%) following ingestion of 16 mL and 37 mL of water by adult and non-adult swimmers, respectively. Site C consistently showed the highest infection probabilities across all pathogens and exposure scenarios. Notably, *Pi* of 24.73%–57.89% for S*almonella* sp., 15.99%–60.58% for *Shigella* sp., and 19.50%–42.42% for *V. cholerae* were observed across all exposure scenarios.

**TABLE 4:** Estimated probability of infection (*Pi*) based on *Salmonella* sp., *Shigella* sp., and *Vibrio cholerae* concentrations in each water sample collected from the Jukskei River across five sampling sites (A–E)^*a*^

Organism	Site	Single exposure			Multiple exposure (*n* = 52)		
		1 mL	16 mL	37 mL	1 mL	16 mL	37 mL
		Mean ± SD			Mean ± SD		
*Salmonella* sp.	A	8.19 ± 7.88	31.02 ± 16.18	39.44 ± 17.00	80 ± 30.58	98 ± 7.07	100 ± 1.43
	B	8.86 ± 12.07	27.45 ± 11.19	36.75 ± 11.45	83 ± 22.55	100 ± 0.38	100 ± 0.01
	C	24.73 ± 17.37	50.78 ± 18.55	57.89 ± 17.18	94 ± 18.68	100 ± 0.13	100 ± 0.00
	D	17.53 ± 15.25	42.96 ± 18.76	50.97 ± 17.23	90 ± 16.86	100 ± 0.01	100 ± 0.00
	E	2.57 ± 4.49	14.73 ± 13.54	21.51 ± 15.91	45 ± 33.9	88 ± 24.49	94 ± 14.51
*Shigella* sp.	A	7.85 ± 11.09	30.32 ± 19.58	41.48 ± 24.66	73 ± 32.87	98 ± 6.13	100 ± 1.02
	B	4.91 ± 4.41	27.81 ± 13.43	38.17 ± 13.92	76 ± 25.56	100 ± 0.08	100 ± 0
	C	15.99 ± 4.42	51.33 ± 5.35	60.58 ± 4.56	100 ± 0.28	100 ± 0.00	100 ± 0
	D	9.50 ± 10.12	33.68 ± 21.09	42.43 ± 22.28	75 ± 35.58	96 ± 12.02	99 ± 4.17
	E	5.80 ± 5.06	29.34 ± 15.75	39.18 ± 16.86	75 ± 29.77	99 ± 3.56	100 ± 0.35
*Vibrio cholerae*	A	1.44 ± 2.22	10.5 ± 13.34	15.41 ± 18.06	32 ± 38.78	54 ± 49.36	57 ± 50.25
	B	2.38 ± 5.20	10.82 ± 17.72	14.37 ± 22.02	28 ± 41.73	39 ± 46.97	43 ± 47.6
	C	19.50 ± 27.61	36.45 ± 32.30	42.42 ± 32.89	66 ± 43.13	79 ± 39.45	82 ± 38.49
	D	17.06 ± 23.96	31.84 ± 32.53	37.13 ± 33.77	57 ± 44.35	71 ± 45.04	73 ± 44.47
	E	1.47 ± 2.90	9.57 ± 13.81	13.9 ± 18.29	28 ± 37.22	49 ± 47.77	53 ± 48.29

^*a*^ *Pi* values are expressed as percentages and summarized as mean ± SD for single and multiple exposures. 1 mL = ingested during baptisms; 16 mL = ingested by an adult swimmer; 37 mL = ingested by a non-adult swimmer.

Multiple weekly exposures increased the *Pi*, regardless of the ingested water volume (Table 4). For *Salmonella* sp., *Pi* ranged between 45% and 94% with weekly ingestion of 1 mL, with site C reaching a *Pi* of 94% and increasing up to 100% with ingestion of 16 mL, and 37 mL (sites B, C, and D). Similarly, *Pi* with *Shigella* sp. reached 100% with multiple exposures to 1 mL of water from site C, and for adult swimmers across sites C and D. For non-adult swimmers, the *Pi* reached 100% at sites A, B, C, and E based on multiple exposures to 37 mL of water (Table 4). Although *V. cholerae* posed a lower infection risk compared to *Salmonella* and *Shigella*, the *Pi* still exceeded the World Health Organization’s (WHO) acceptable threshold of 0.01%. From a single exposure to 1 mL of ingested water, the *Pi* with *V. cholerae* ranged from 1.44% to 19.5%, peaking at site C (Table 4). For casual swimmers, *Pi* with *V. cholerae* ranged from 9.57% to 36.45% for adults and 13.9% to 42% for non-adults. Multiple weekly exposures further increased the *Pi* with *V. cholerae* to 79% and 82% for adult and non-adult swimming at sites C.

## DISCUSSION

### Water quality of the river

The Jukskei River is one of the largest rivers in Johannesburg, South Africa. It holds immense significance for both the environment and the communities that live on its banks, serving as a sacred site for cultural and religious rituals, such as baptisms. However, this river is a toxic cocktail of sewage and pollution, largely due to the anthropogenic activities of the growing population, as well as industrial and sewage discharge on account of an ailing infrastructure (24). This study aimed to evaluate the water quality and the extent of microbial contamination in this urban South African river and determine the public health risk associated with exposure to enteric pathogenic bacteria in the water.

The water quality of the river was determined by measuring pH, temperature, and TDS, respectively. In addition, indicator organisms were used to assess the microbial contamination of water using total coliform and *E. coli* counts. Surprisingly, most of the analyzed physico-chemical parameters were within acceptable limits according to the SAWQ guidelines for domestic and recreational. The pH of the river was within the target water quality range for domestic use (25). However, when comparing the pH levels measured in our studies to previously published data, there is a notable decrease in the pH over the past 13 years, from a mean pH value of 8.30 reported in a study published in 2010 (26) to a mean of 7.98 observed in a 2020 (27), the pH decreased to 7.31 in a study published in 2021 (28) and in our study an even lower pH of 7.29 was noted. This observed decline in pH likely reflects increasing anthropogenic pressures on the river, indicating a shift toward more acidic conditions, which may have implications for aquatic life and microbial ecology. The temperature of the river water was also within acceptable limits, ranging from 14°C to 22°C (mean temperature of 18°C) across the rainy and dry seasons. Interestingly, in a previous study by Hoorzook et al. (28), a mean temperature of 15.9°C was noted, much lower than the temperature recorded in our study, suggesting that the river is getting warmer. This is indicative of the effects of climate change and aligns with a broader climate change impact observed in a study that analyzed climate data from 1976 to 2019 in the Olifants River Catchment, South Africa, which observed an increase in surface temperatures (29). This is concerning as warmer river water can result in the reduction of oxygen levels, which disrupts the ecosystem, promoting algal bloom, leading to poor water quality (30, 31).

The mean TDS in the river was 160 mg/L, higher than the values reported in a 2010 study, which ranged from 10 to 104 mg/L (26), but lower than the values (262 to 302 mg/L) previously reported in a 2021 study (28). The variability of TDS values highlights the importance of determining local conditions for site-specific TDS concentrations (25). According to DWAF, the TDS in rivers should not differ by more than 15% in any season. A similar study by Teffo (27) had the highest TDS of 349 mg/L. This deviated from our study and the stipulated 15% change by the DWAF, suggesting huge discrepancies in TDS values. While Teffo (27) measured the TDS on-site using a portable pH/EC/TDS meter, we measured the TDS in the laboratory, further accounting for the discrepancies.

### Microbial burden and public health implications

For environmental water surveillance and drinking water safety assessment, *E. coli* and TC are widely used as indicator organisms. In this study, both indicators revealed high levels of fecal contamination in the Jukskei River, with TC and *E. coli* counts above 2,000 CFU/100 mL and 400 CFU/100 mL, respectively. *E. coli* is a commensal organism found in the intestines of humans and other warm-blooded animals, whereas other coliforms may originate from soil or vegetation. Therefore, the presence of *E. coli* in water is an indicator of possible fecal contamination from human or animal sources. Several studies have validated this association; specifically, Mothiba et al. (32) and Fejfar et al. (33) confirmed *E. coli* as a reliable marker of fecal pollution. Similar findings were reported in northern Sweden and Pakistan, where *E. coli* detection in water sources was consistently linked to fecal contamination (34, 35).

In a press report, Turton (36) reported that about 4.2 billion litres of raw sewage are discharged daily into many rivers in South Africa, including the river in this study (36). This unpleasant situation is exacerbated during periodic flooding, particularly during the rainy season. This increases the river flow rate and negatively impacts the neighboring communities’ health, hygiene, and sanitation. Moreover, the detection of *E. coli* is an indicator of possible fecal contamination and suggests the potential presence of pathogenic microorganisms. High coliform counts were observed in the rainy seasons (higher temperatures) compared to the dry seasons. In contrast, high counts of *E. coli* were observed in the dry seasons (lower temperatures) compared to the rainy seasons. Our findings corroborate results from a study by An et al. (37) that reported low *E. coli* densities in the summer, which they attributed to the lower loading of fecal material during summer, more vigorous grazing by protozoa, and less viability in warm water. Similarly, densities of total coliforms greatly increased in the summer at elevated temperatures (37). When comparing the counts of the indicator organisms across the five sampling sites, high coliform and *E. coli* counts were observed in sites C and D, with lower counts in site E. The river passes along geographically distinct sites characterized by varying population densities and socio-economy. Site C is located within a densely populated urban informal settlement and impoverished community, with high unemployment, poor infrastructure, and service delivery, as well as large migrant communities facing limited economic opportunities. Microbial contamination, more specifically *E. coli,* resulting from informal settlements along riverbanks, has been well documented in various studies (38–40). Site D is located less than 2 km downstream of site C; thus, it receives contaminated effluent from site C. In contrast, site E, 15 km downstream from site C, runs alongside affluent neighborhoods, with well-maintained resources, facilities, infrastructure, private security, and exclusive golf estates. Notably, the TC and *E. coli* counts in site E were less than 2,000 CFU/100 mL and 400 CFU/100 mL, respectively, in both the rainy and dry seasons. Nevertheless, the SAWQ guidelines for recreational use indicate that the risk of contracting gastrointestinal illness is increased when counts are above 2,000 CFU/100 mL and 400 CFU/100 mL for TC and *E. coli*, respectively. Noticeable gastrointestinal health effects are expected in the swimmers and bather population when counts are between 600 and 2,000 CFU/100 mL for TC, whereas a slight risk of gastrointestinal effects among swimmers and bathers may be expected when *E. coli* counts are between 130 and 200 CFU/100 mL (41). In terms of agricultural use, irrigation water with *E. coli* counts between 1 and 1,000 CFU/100 mL has an increased risk of contaminating vegetables and other crops, resulting in the transmission of waterborne pathogens to humans (42). Therefore, this urban river is not suitable for recreational and agricultural use as it exceeds the permissible microbial limit.

The presence of *E. coli* indicates the presence of bacterial pathogens such as *Salmonella*, *Shigella, Vibrio cholerae*, *Campylobacter coli*, *Campylobacter jejuni*, *Yersinia enterocolitica,* and pathogenic *E. coli* (9). Indeed, *Salmonella*, *Shigella*, and *Vibrio cholerae* were detected in the river. The bacterial counts were consistent with the geographies of the sampling sites, with sites C and D accounting for staggeringly high levels of microbial contamination compared to site E. Concerningly, these pathogens are among the most significant pathogens with a dire impact on public health. *Salmonella* is the etiological agent of typhoid fever and non-typhoidal salmonellosis and is one of the leading causes of intestinal illness worldwide (43). Its survival and proliferation in water bodies are influenced by pollution, climate variability, and the physicochemical characteristics of water, such as pH (6.5–7.5), elevated temperatures, which accelerate its reproduction and dissemination, as well as dissolved oxygen, as *Salmonella* can survive in low-oxygen environments (44). These conducive conditions were notably observed in the river, suggesting that the river constitutes a favorable ecological niche for the survival, growth, and proliferation of *Salmonella* sp. Therefore, the sustained presence of this pathogen represents a significant public health concern.

*Shigella* is a highly virulent pathogen that causes bacterial dysentery and continues to carry a significant burden of disease worldwide, more especially in Asia and Africa (45). *Shigella* sp. have been detected in other South African rivers (14, 46). Interestingly, Ekwanzala et al. (46) also reported high levels of *Salmonella* and *Shigella* in an urban river in Pretoria, South Africa. Similar to our study, *Shigella* counts were much higher than the observed *Salmonella* counts (46). While cholera is not endemic in South Africa, there have been recurrences of cholera outbreaks in recent years (47), with the Jukskei River implicated in some cholera illnesses, including an outbreak that occurred in 2023 in the Gauteng Province, involving an imported case following travel to Malawi (48).

The presence of these enteric pathogens in this urban river poses a serious public health risk, as the river is utilized by communities for baptisms and other religious activities, where unintentional ingestion of water can occur, leading to severe disease and outbreaks. The risk of infection varies by pathogen, species, and strain. *Salmonella* typically requires an infectious dose of 10³ to 10⁵ cells (49), while cells as low as 10 to 100 for *Shigella* are enough to cause disease (50). The infectious dose is much higher in *Vibrio cholerae*, ranging from 10^3^ to 10¹¹ cells, depending on the host conditions, such as gastric acidity (51). In our study, the QMRA was used to evaluate the public health risks associated with ingestion of 1 mL, 16 mL, and 37 mL of the water from this urban river. The results revealed that the risk of infection is high across all sampling sites and exceeds the WHO’s acceptable risk limit of 10^−4^ or 0.01% (52). Moreover, the probability of infection with *Salmonella* and *Shigella* is higher, particularly in sites C and D, while the risk from *Vibrio cholerae* is comparatively lower. The high infection risk recorded with *Salmonella* and *Shigella* in site C, assuming a single exposure to 1 mL of the river water, highlights the significant health risk posed by the river to the informal settlement along the riverbanks that is often inundated with contaminated water when the river swells during periodic flooding in the rainy season. Concerningly, children from this informal community could be at an even higher risk of infection due to their weak immune system (21). Our findings also revealed that the probability of infection increases with the volume of water ingested as well as with multiple exposures. These results corroborate other published studies that reported a higher risk of infection when large volumes of contaminated water are ingested and upon repeated exposure (19, 21, 53).

One notable limitation of the QMRA is the subjectivity in model and parameter selection, such as the dose-response model used, ingestion volumes, and exposure assumptions, which can affect the estimated risk. Nevertheless, our findings highlight the urgent need for improved water quality management and public health interventions for the river to protect vulnerable communities from the risks associated with this contaminated water. Improvements in water management, infrastructure, proper sewage systems, and flood prevention strategies within impoverished communities are essential to ensure adequate sanitation of the river and the improved health of the surrounding communities.

A key limitation of this study is the absence of an epidemiological investigation screening for *Salmonella*, *Shigella*, and *Vibrio cholerae* in the affected population. Thus, the direct role of the river in local disease transmission could not be determined. Future studies should use the One Health Approach, integrating environmental, clinical, and epidemiological data to better assess the public health impact of microbial contamination in urban rivers like the Jukskei. Additional limitations relate to the microbiological methods used. While the selective media used in this study are widely accepted and compliant with international standards (EN ISO 16140-2:2016, ISO 6579-1:2017, and ISO 21872-1:2017, respectively), they are not perfectly selective. In complex environmental samples with high background flora, non-target organisms may grow and mimic the appearance of target pathogens, potentially affecting the accuracy of presumptive counts. Although XLD agar has a higher efficiency than Deoxycholate Citrate Agar for isolating *Shigella* sp*.*, it is less selective than media such as Hektoen Enteric and Salmonella-Shigella agar. These highly selective media are preferred when high background flora is expected, but may be too stringent for stressed *Shigella* strains, potentially reducing recovery rates (54). Given the environmental complexity of the Jukskei River, XLD was selected to balance selectivity and recovery efficiency. Furthermore, due to time and resource constraints, only a few colonies per plate were confirmed using PCR for each microorganism. While this approach is consistent with practices in environmental microbiology studies, it can affect the accuracy of pathogen quantification. Future studies should consider confirming a larger number of colonies to improve quantification accuracy and better reflect the microbial complexity of environmental water sources. Moreover, this study did not account for the presence of viable but non-culturable (VBNC) organisms, which are commonly found in environmental samples and may evade detection by culture-based methods. Incorporating molecular techniques such as viability PCR for bacterial quantification in future work could enhance detection sensitivity and provide a more comprehensive assessment of pathogen presence.

### Conclusion

This study represents, to our knowledge, the first attempt to quantitatively assess the microbial risks associated with contamination of the Jukskei River, an urban waterway in South Africa. Although physicochemical parameters such as pH and temperature were within the DWAF guidelines, microbiological analysis revealed extensive fecal contamination, possibly from sewage discharged into the river due to a lack of proper infrastructure, as well as poor management of the existing structural resources, particularly in the communities along the riverbanks. Elevated counts of *E. coli* and TC, along with the detection of *Salmonella*, *Shigella*, and *Vibrio cholerae*, indicate significant public health risks, particularly for communities residing along informal settlements adjacent to the river. The presence of these enteric pathogens further highlights the underlying socioeconomic disparities within the country and underscores the effects of limited infrastructure and overpopulation on the health of the environment. The estimated probability of infection from exposure to river water exceeded acceptable thresholds, with higher risk observed at sites near informal settlements. These findings underscore the urgent need for targeted interventions to improve water quality and mitigate health risks in vulnerable populations. This study contributes to the growing body of evidence on urban waterway pollution and its implications for public health. Future research should adopt a One Health surveillance framework, integrating environmental, clinical, and epidemiological data to enable early detection of enteric pathogens and inform more effective public health interventions in South Africa.

## Data Availability

All the relevant data are contained within the paper. Raw data are available in the supplemental material.

## SUPPLEMENTAL MATERIAL

Graphical abstract aem.01566-25-s0001.tif Visual depiction of the study findings.

Supplemental material aem.01566-25-s0002.docx Tables summarizing the geographic characteristics of the sampling sites and the raw bacterial count data expressed as log CFU/100 mL.

ASM does not own the copyrights to Supplemental Material that may be linked to, or accessed through, an article. The authors have granted ASM a non-exclusive, world-wide license to publish the Supplemental Material files. Please contact the corresponding author directly for reuse.
