# Supplementary figures and images for: A river in crisis: water quality, microbial burden, and public health implications of a South African urban river
Source: Appl Environ Microbiol. 2025 Oct 8;91(11):e01566-25. doi: 10.1128/aem.01566-25 (PMC12628798; doi:10.1128/aem.01566-25)

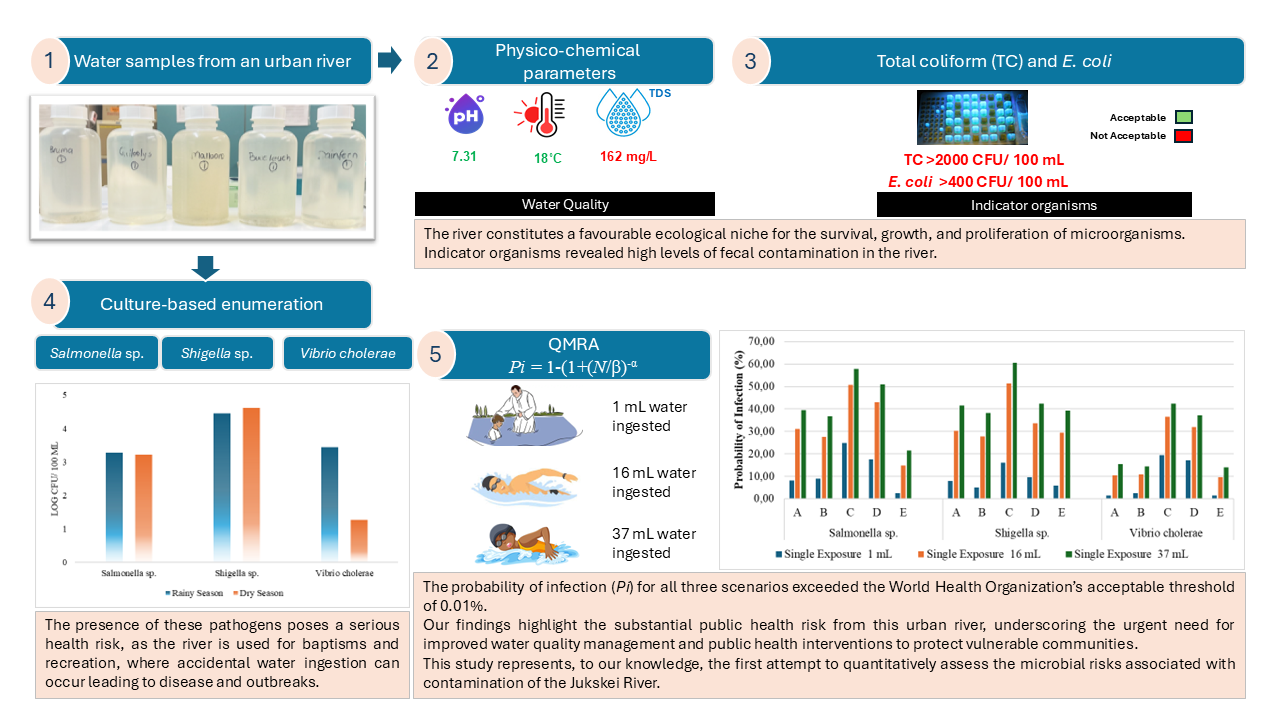

Supplement: Graphical abstract — Visual depiction of the study findings. [file aem.01566-25-s0001.tif]
