# Supplementary material for: A river in crisis: water quality, microbial burden, and public health implications of a South African urban river
Source: Appl Environ Microbiol. 2025 Oct 8;91(11):e01566-25. doi: 10.1128/aem.01566-25 (PMC12628798; doi:10.1128/aem.01566-25)
Supplement: Supplemental material — Tables summarizing the geographic characteristics of the sampling sites and the raw bacterial count data expressed as log CFU/100 mL. [file aem.01566-25-s0002.docx]

Table S1. The characteristics of the five sampling sites along the Jukskei River.

| **Sites** | **Coordinates** | **Site Description** |
| --- | --- | --- |
| **A** | 26,18147°S. 28,10237°E | A suburb known as the gateway to Johannesburg. home to numerous hotels. business headquarters. office parks. and motor dealerships. The headwaters of the Jukskei River flow into Bruma Lake. contributing to its water system. |
| **B** | 26,16475°S. 28,13029°E | A recreational park home to a diverse array of bird species. including the African Darter. Yellow-billed Duck. and Egyptian Goose. The park also attracts visitors who use the river for cultural and religious practices. such as baptisms and initiations. |
| **C** | 26,08208°S. 28,10873°E | A suburb bordering one of the most densely populated and poorest informal urban settlements in Johannesburg. which is situated along the banks. is prone to flooding during the rainy season. The community also uses the river for cultural and religious practices. |
| **D** | 26,06715°S 28,10767°E | A suburb situated along the Jukskei River 1. 66 km from Site C. |
| **E** | 25,98690°S 27,99731°E | A prestigious golfing estate in greater Johannesburg. renowned for its unparalleled beauty and recreational offerings. including nature trails. parks. and sports facilities. The Jukskei River runs through this well-maintained estate. |

Table S2: Raw data of the *Salmonella* sp., *Shigella* sp., and *Vibrio cholerae* counts in Log CFU/ 100 mL along with Mean±SD for each site.

| ***Salmonella sp.*** | | | | ***Shigella sp.*** | | | ***Vibrio cholerae*** | | |  |
| --- | --- | --- | --- | --- | --- | --- | --- | --- | --- | --- |
|  |  |  |  |  |  |  |  |  |  |  |
| **Sites** | **Rainy** | **Dry** | **Mean±SD** | **Rainy** | **Dry** | **Mean±SD** | **Rainy** | **Dry** | **Mean±SD** |  |
| A | 3.13 | 2.82 | 3.14±0.72 | 3.49 | 4.48 | 4.38±0.78 | 1.95 | 3 | 1.82±1.68 |  |
|  | 2.82 | 3.19 |  | 3.04 | 4.08 |  | 3.18 | 0 |  |  |
|  | 3.3 | 3.5 |  | 4.78 | 5.04 |  | 3.7 | 0 |  |  |
|  | 3.16 | 4.08 |  | 5.95 | 3.7 |  | 2.6 | 0 |  |  |
|  | 2.18 | 1.63 |  | 4.56 | 4.9 |  | 3.9 | 0 |  |  |
|  | 3.73 | 4.09 |  | 4.04 | 4.48 |  | 0 | 3.48 |  |  |
| B | 3.22 | 3.05 | 3.0±0.44 | 4.6 | 3.7 | 4.32±0.46 | 0 | 0 | 1.53±1.78 |  |
|  | 2.93 | 2.18 |  | 4.4 | 4.48 |  | 3.48 | 0 |  |  |
|  | 2.87 | 3 |  | 3.78 | 3.78 |  | 3.2 | 0 |  |  |
|  | 2.79 | 3.34 |  | 4.78 | 3.9 |  | 4.46 | 0 |  |  |
|  | 3.69 | 2.5 |  | 5.14 | 4.73 |  | 3.85 | 1.48 |  |  |
|  | 3.1 | 3.74 |  | 4.48 | 4.11 |  | 0 | 1.85 |  |  |
| C | 4.15 | 3.87 | 4.04±0.85 | 5.34 | 5.18 | 5.12±0.19 | 3.32 | 4.04 | 3.57±2.15 |  |
|  | 4.3 | 3.32 |  | 5.11 | 5.15 |  | 6.32 | 2.95 |  |  |
|  | 4.86 | 2.3 |  | 5 | 5.34 |  | 5.61 | 0 |  |  |
|  | 3.32 | 5.38 |  | 4.95 | 5.2 |  | 4.6 | 0 |  |  |
|  | 4.14 | 3.43 |  | 5.26 | 4.78 |  | 6.6 | 1.95 |  |  |
|  | 4.5 | 4.87 |  | 5.32 | 4.85 |  | 3.78 | 3.7 |  |  |
| D | 3.8 | 3.39 | 3.69±0.79 | 3.48 | 5.6 | 4.46±0.86 | 3.04 | 3.48 | 3.13±2.24 |  |
|  | 4.79 | 2.54 |  | 3.48 | 4.48 |  | 6.11 | 1.9 |  |  |
|  | 4.9 | 3.18 |  | 5.23 | 5 |  | 5.49 | 0 |  |  |
|  | 2.73 | 3.67 |  | 2.78 | 4.36 |  | 5.36 | 0 |  |  |
|  | 2.77 | 4.28 |  | 5.51 | 4.78 |  | 5.18 | 0 |  |  |
|  | 3.94 | 4.28 |  | 4.23 | 4.59 |  | 3.6 | 3.4 |  |  |
| E | 3.82 | 2.88 | 2.35±0.78 | 3.95 | 5.11 | 4.35±0.59 | 0 | 3.18 | 1.72±1.65 |  |
|  | 2.81 | 1.95 |  | 3.18 | 4.7 |  | 4.11 | 1.7 |  |  |
|  | 1.04 | 2.61 |  | 4.23 | 5 |  | 3.49 | 0 |  |  |
|  | 1.18 | 2.41 |  | 4.6 | 4.91 |  | 2.85 | 0 |  |  |
|  | 2.08 | 1.94 |  | 4.04 | 4.15 |  | 3.36 | 1.9 |  |  |
|  | 2.38 | 3.04 |  | 4.7 | 3.6 |  | 0 | 0 |  |  |
| **Mean±SD/Year** | 3.28±0.97 | 3.22±0.88 |  | 4.45±0.79 | 4.61±0.53 |  | 3.44±1.92 | 1.27±1.50 |  |  |
| *p-value* | *0.3714* | | | *0.8269* | | | *0.0001* | | |  |
